# Sex-specific deficits in neurite density and white matter integrity are associated with targeted disruption of exon 2 of the *Disc1* gene in the rat

**DOI:** 10.1038/s41398-019-0429-2

**Published:** 2019-02-11

**Authors:** Brian R. Barnett, Maribel Torres-Velázquez, Sue Y. Yi, Paul A. Rowley, Emily A. Sawin, C. Dustin Rubinstein, Kathleen Krentz, Jacqueline M. Anderson, Vaishali P. Bakshi, John-Paul J. Yu

**Affiliations:** 1 Neuroscience Training Program, Wisconsin Institutes for Medical Research, University of Wisconsin–Madison, Madison, WI 53705 USA; 2 Department of Biomedical Engineering, College of Engineering, University of Wisconsin–Madison, Madison, WI 53706 USA; 3 Department of Radiology, University of Wisconsin School of Medicine and Public Health, Madison, WI 53705 USA; 4 Biotechnology Center, University of Wisconsin–Madison, Madison, WI 53706 USA; 5 Department of Psychiatry, University of Wisconsin School of Medicine and Public Health, Madison, WI 53705 USA

## Abstract

Diffusion tensor imaging (DTI) has provided remarkable insight into our understanding of white matter microstructure and brain connectivity across a broad spectrum of psychiatric disease. While DTI and other diffusion weighted magnetic resonance imaging (MRI) methods have clarified the axonal contribution to the disconnectivity seen in numerous psychiatric diseases, absent from these studies are quantitative indices of neurite density and orientation that are especially important features in regions of high synaptic density that would capture the synaptic contribution to the psychiatric disease state. Here we report the application of neurite orientation dispersion and density imaging (NODDI), an emerging microstructure imaging technique, to a novel *Disc1* svΔ2 rat model of psychiatric illness and demonstrate the complementary and more specific indices of tissue microstructure found in NODDI than those reported by DTI. Our results demonstrate global and sex-specific changes in white matter microstructural integrity and deficits in neurite density as a consequence of the *Disc1* svΔ2 genetic variation and highlight the application of NODDI and quantitative measures of neurite density and neurite dispersion in psychiatric disease.

Neuroimaging has uncovered differences in the structural and functional organization of the brain across a broad spectrum of neuropsychiatric disorders^1^. With the development of quantitative neuroimaging techniques such as diffusion tensor imaging (DTI), these efforts have centered on white matter microstructure as a means to explore the biological basis of brain microstructure and connectivity^2,3^. Explorations of brain disconnectivity have focused on the disruption of axonal projections, myelination, and orientation of white matter tracts between cortical areas more so than on disruption of synaptic changes, which are largely occult with standard DTI techniques^4^. The ability to interrogate these synaptic changes as well as microstructural features outside of large white matter tracts has spurred the earnest development of new advanced diffusion weighted imaging methodologies. These new methods include quantitative multi-compartment diffusion weighted imaging techniques such as neurite orientation dispersion and density imaging (NODDI) that represent an extension of single-compartment diffusion tensor models like DTI. Whereas quantitative indices of DTI such as fractional anisotropy (FA) are able to capture microstructural features but are inherently nonspecific, multi-compartment diffusion techniques such as NODDI are able to model water diffusion across multiple compartments, thus enabling more granular microstructural information such as neurite density and orientation that are important features in these regions of higher synaptic density.

Recent work has begun to uncover evidence for the unanticipated genetic^5–7^, molecular^8^, and neurostructural^9^ convergence of several psychiatric diseases including autism spectrum disorder (ASD), schizophrenia, bipolar disorder, and major depression. These neurobiological data dovetail into new dimensional frameworks of psychiatric disease on the basis of shared disease comorbidity and neurobiology and bolsters the development of the Research Domain Criteria from the National Institute of Mental Health. *DISC1* is one such gene that stands at the intersection of numerous psychiatric diseases. As with other genetic variants that have been shown to confer an increased risk for disease^10^, the balanced chromosomal t(1;11)(q42.1;q14.3) translocation of the *DISC1* gene has been implicated in psychiatric illnesses including schizophrenia and developmental disorders^11–13^, bipolar disorder^13^, autism spectrum disorder^14^, and major depressive disorder^15^. *DISC1* has not shown promise as a common risk gene for schizophrenia; however, research exploring the function of *DISC1* in early brain development still represents an avenue to understand a key molecular driver in the neuropathogenesis of mental illness^16,17^. Towards these ends, several groups have generated animal models of *Disc1* towards understanding the unique molecular neuropathogenesis of psychiatric disease that arises from this genetic locus. These include models with dominant-negative *Disc1* expression and models with ENU mutagen-induced point mutations^18,19^. Loss-of-function *Disc1* murine models have also been generated including a murine model lacking exons 2 and 3 of the *Disc1* gene that displays abnormalities in sensorimotor gating, impulsive behavior, and cognitive impairments centering around repetitive and compulsive-like behaviors^20,21^. Other models such as a murine locus impairment model with a deletion covering exons 1, 1b, 2, and 3 from the *Disc1* gene have also been produced^22^.

To expand the repertoire of translational *Disc1* models available, we sought to generate a new rat *Disc1* model that would be amenable to detailed behavioral, neuropharmacologic, and translational neuroimaging studies. Numerous splice variants of the *DISC1* gene (with more than 50 splice variants reported in humans^23^) in addition to many possible unknown splice variants^24^ limits the straightforward generation of a knockout model; however, the biological consequences of early DISC1 truncation are associated with and are seen in patients with schizophrenia^25^ and several models recapitulating early truncation of the major isoform of DISC1 represent some of the most illuminating animal models of *DISC1* neuropathophysiology^22,24,26^. To further our understanding of these short variants in the physiological function of DISC1, we report herein the generation of a novel rat short genetic variant model of *DISC1* truncation (*Disc1* svΔ2) that lacks exons 2-13 following targeted deletion with CRISPR/Cas9. Our *Disc1* svΔ2 rat model demonstrates the impact of *Disc1* truncation in global measures of white matter structural integrity with DTI and the global deficits in neurite density attributable to *Disc1* that are found not only in regions of decreased white matter structural integrity but also in previously uncharacterized gray matter structures. These findings highlight potential areas of shared comorbidity in ASD, schizophrenia, bipolar disorder, and major depression that would be seen in a dimensional approach to neuropsychiatric disease and also speak to the exciting potential and utilization of multi-shell diffusion weighted imaging to capture new quantitative neuroimaging metrics in neuropsychiatric illness.

## Materials and methods

### Model generation

Animals were housed and cared for in an AAALAC-accredited facility and all animal experiments were conducted in accordance with local IACUC-approved protocols. Utilizing the CRISPR-Cas9 genome-editing technique, the second coding exon of the rat *Disc1* gene encoding amino acids 19-342 (RefSeq transcript ENSRNOT00000057945.4) was targeted to generate a nonsense mutation. Two highly-specific target sequences were selected ([1] ATGCCACGTCCGATCTCAGCGGG; [2] TCAACGGGGCCATTCGACGCCGG). All predicted off-targets for both targets varied by at least 3 nucleotides and no single predicted off-target had an activity prediction score (CFD) higher than 0.5^27,28^. An in vitro transcription template was generated by overlap-extension PCR with one oligo carrying a 5′ T7 adapter, the target sequence, and a portion of the common gRNA sequence, and the other oligo carrying the antisense common gRNA sequence. Following column-purification, the in vitro transcript was transcribed with the MEGAshortscript kit (ThermoFisher), and the resultant gRNA was cleaned with the MEGAclear kit (ThermoFisher), purified with ammonium acetate, washed with 70% ethanol, and resuspended in injection buffer (10 mM Tris-HCl, 0.1 mM EDT, pH 7.4). One-cell fertilized Sprague Dawley (SD) embryos were microinjected with a mixture of both gRNAs (25 ng/uL each) and Cas9 protein (PNA Bio, 40 ng/uL), and then implanted into pseudopregnant female Sprague-Dawley (SD) recipients. Resultant pups were genotyped at weaning by PCR, amplifying the targeted region. Founders yielded an approximate 389 bp fragment indicating the successful excision between the two target sites. Putative excision fragments were gel-purified and sequenced. Those founders bearing deletions causing a non-sense mutation and early termination of translation were bred to SD mates. F1s carrying the excision allele were twice verified by both PCR and DNA sequencing.

### Subjects

Outbred control male and female Sprague-Dawley rats (300–325 g, Charles River, Worcester, MA, USA) and *Disc1 svΔ2* male and female rats were pair housed in clear cages and were maintained under a 12:12 h light:dark cycle in humidity- and temperature-controlled rooms with *ad libitum* access to food and water. Sprague-Dawley pregnant dams ordered from Charles River arrived at the housing facility, after which all Sprague-Dawley and *Disc1 svΔ2* male and female rats used in our data analyses were born, weaned, and matured to adulthood in the same housing facility. Animals were acclimated to housing conditions for seven days prior to experimental manipulation. To generate the experimental *Disc1* svΔ2 animals, all *Disc1 svΔ2* male and female animals were generated from *Disc1 svΔ2* male-female homozygous pairings and subsequently genotyped to confirm genetic background. For Western blot analysis, brain tissue was homogenized in lysis buffer (20 mM Tris- HCl, 137 mM NaCl, 10% glycerol, 1% TritonX-100, 2 mM EDTA, pH 8); containing protease inhibitors (Roche, Basel, Switzerland) then centrifuged at 14,000×*g* for 20 min. Protein samples were separated by SDS polyacrylamide gel electrophoresis in 10–12% gradient gels and transferred onto polyvinylidene difluoride membranes (0.45 μm). The membranes were then incubated with the primary anti-DISC1 (1:1000) antibody at 4 °C overnight (rabbit DSMS/D22 anti-DISC1 598–785, purified on recombinant rat DISC1 (598–785), a generous gift from Carsten Korth, Universität Düsseldorf, Germany). The membranes were then incubated with horseradish peroxidase-conjugated secondary antibodies (1:10000) for 60 min (Santa Cruz Biotechnology, Dallas, TX, United States). Target bands were detected and quantified using a fluorescence scanner (Bio-Rad, Hercules, CA, United States).

### Imaging methodology

#### Data acquisition

Ex-vivo imaging methods were used to examine the structural differences in our *Disc1 svΔ2* genetic model (*n* = 6 for both males and females) as compared to age and sex-matched controls (*n* = 7 for control males and *n* = 6 for control females). Sample sizes for ex-vivo neuroimaging experiments were determined according to power analyses and from previous animal imaging studies^29^. All samples within each genotype (*Disc1 svΔ2* and wild-type SD) were randomly selected for imaging. All imaged animals were used in subsequent analyses. Investigators were not blinded to group allocation during data collection or data analysis; subject identification does not impact quantitative measures of diffusion weighted imaging that were subsequently collected. At postnatal day 84, animals were anesthetized with isoflurane and transcardially perfused with 4% paraformaldehyde (PFA) following an initial wash with PBS. Following dissection from the cranial vault, fixed brains were stored in 4% PFA until imaging and rinsed in 0.9% saline for 48 h prior to imaging to minimize attenuating effects of fixative on MRI signal. The brains were placed in a custom-built holder and immersed in Fluorinert (FC-3283, 3 M, St. Paul, MN, USA). For ex-vivo DTI acquisition, brains were imaged using a 4.7-T Agilent MRI system and 3.5-cm diameter quadrature volume RF coil. Multi-slice, diffusion-weighted, spin echo images were used to acquire 10 non-diffusion weighted images (b = 0 s*mm^-2^) and 75 diffusion-weighted images (25: b = 800 s*mm^-2^, 50: b = 2000 s*mm^−2^), using non-colinear weighting directions. Other imaging parameters were TE/TR = 24.17/2000-ms, FOV = 30 × 30 mm^2^, matrix = 192 × 192 reconstructed to 256 × 256 for an isotropic voxel size of 0.25-mm over two signal averages.

#### Data preprocessing

Raw data files were converted to NIfTI (Neuroimaging Informatics Technology Initiative) format. FMRIB Software Library (FSL) was used to correct for eddy current artifacts and to fit the diffusion tensors to each volume series. The FSL DTI output volumes were converted to NIfTI tensor format for use with the DTI-TK software package. DTI-TK^30^ was used to estimate a study-specific tensor template, which was used as a target to which each subject tensor volume was spatially normalized.

#### Tract-based spatial statistics

A tract-based spatial statistics (TBSS) processing chain was adapted by replacing the standard TBSS registration (FSL’s FNIRT) with the DTI-TK registration routine. The TBSS pipeline was applied utilizing the recommended parameters in FSL. An FA threshold of 0.2 was applied for the creation of the skeleton and a permutation test with *n* = 252, corrected for multiple comparisons and threshold-free cluster enhancement was implemented with FSL’s Randomize to compare each of the experimental groups to the control group, with *p* *<* .05 as threshold for significance. As all neuroimaging experiments were performed ex-vivo, a random sample of brains was selected for repeat ex-vivo imaging. These data were then processed again as described above and a TBSS analysis was then performed on the repeat scanned brain as compared to the original brain scan data with no significant interscan differences identified on a voxel-wise basis.

#### Region of interest analysis

The UNC P72 Rat Atlas was normalized to subject common space and masked with predefined regions-of-interest (ROIs)^31^. Diffusion measures for all regions of interest from the atlas were extracted. Following automated volumetric segmentation of the brain, mean values of both diffusion and neurite indices were computed within six ROIs (hippocampus, external capsule, basal ganglia, internal capsule, neocortex, and corpus callosum) in each hemisphere for each individual subject. These ROIs were selected based on their relevance to mental illness for both major white matter and gray matter regions. Two-tailed, two-sample, and unequal variance Student’s *t*-Test was performed comparing fractional anisotropy (FA), axial diffusion (AD), radial diffusion (RD), mean diffusivity (MD) (MD = (1/3)(TR); TR = trace diffusivity), neurite density index (NDI), and orientation dispersion index (ODI) mean values in *Disc1 svΔ2* animals against age-sex-matched controls. Raw *p*-values were reported and adjusted *p*-values were calculated using the Benjamini-Hochberg false discovery rate (FDR) correction (FDR = .05).

### Startle chambers

Startle chambers (San Diego Instruments, San Diego, CA) containing a Plexiglas cylinder resting inside a ventilated and illuminated sound-attenuating cabinet with a high-frequency loudspeaker to produce all acoustic stimuli were used as previously described previously^32^.

#### Startle and PPI testing

Prepulse inhibition testing took place on postnatal day 84 for all subjects. The test session consisted of background noise (65 dB) presented alone for 5 min and remained on for the length of the session, followed by presentation (in a pseudo-random order) of pulse-alone trials (40-ms, 120-dB broadband bursts), prepulse + pulse trials (20-ms noises that were 3, 9, or 15 dB above the background noise and were presented 100 ms before the onset of the 120-dB pulse), and no stimulus trials (only the background noise). The session included 16 each of the 3, 9, and 15-dB prepulse + pulse trials, 16 pulse-alone trials and 16 no stimulus trials. Four pulse-alone trials were presented at the beginning and the end of the session to ensure that startle magnitude was stable during the portion of the session when PPI was measured; these pulse-alone trials were excluded from the calculations of startle magnitude and %PPI. Investigators were not blinded to group allocation during data collection or data analysis. Subject identification does not impact quantitative measures of prepulse inhibition that were subsequently collected. Following pre-established procedures, animals that did not demonstrate a mean startle response of at least 100 μV (as measured by the piezoelectric sensor in the prepulse inhibition apparatus) were excluded from prepulse inhibition data analysis. These animals did not exhibit enough of a startle response to utilize as a baseline for calculating percent prepulse inhibition values on each of the prepulse trials. For behavioral testing, the sample sizes were as follows: control male group, *n* *=* 24, control female group, *n* = 18, *Disc1 svΔ2* male group, *n* *=* 15, and *Disc1 svΔ2* female group, *n* = 15. Sample sizes for prepulse inhibition behavioral assays were calculated according to prior power analyses from co-authors’ previous prepulse inhibition experiments.

### Data analysis

The startle response to the onset of the 120-dB burst was recorded for 100 ms for each pulse-alone trial, prepulse + pulse trial, and from the onset of each no stimulus trial. Two measurements (startle magnitude and %PPI) were calculated from these values for each rat for each of the different treatment conditions. Startle magnitude was the average of the startle responses to all pulse-alone trials. %PPI was calculated as a percent score for each prepulse + pulse trial type: % PPI = 100− ((startle response for prepulse + pulse trial)/(startle response for pulse-alone trial)). Latency to startle was calculated by the startle chamber software from the time point of trial onset to when the subject first made a movement that triggered the piezoelectric sensor. Latency to maximum startle was calculated from the time point of trial onset to when the subject made a movement that resulted in the maximal piezoelectric sensor response. For behavioral analyses, pre-pulse inhibition, maximum startle, latency to startle, latency to maximum startle, and habituation data were subjected to three-way ANOVA with genotype and sex as between-subjects factors and trial type or habituation block as repeated-measures factors. Tukey’s honest significant difference post hoc comparison was used to detect differences at the *p* < .05 level. The behavioral data analyzed meets the assumptions of normality and homogeneity of variances.

## Results

### Generation of the rat *Disc1 svΔ2* genetic model

**Fig. 1:**
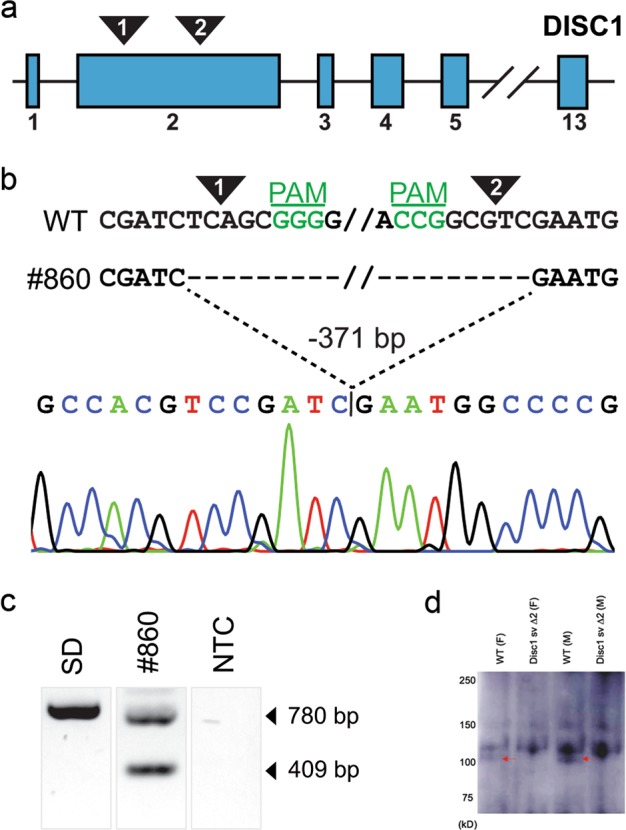
Generation of the rat *Disc1 svΔ2* model. **a** The selected strategy was a redundant 2-target CRPSPR/Cas9 approach (low transcript complexity, large targetable N-terminus exon). Two target sites in exon 2 were selected in the second coding exon. **b** Expected target 1 and 2 cleavage sites are indicated (arrowhead), and target 1 and 2 PAMs (green) are on the sense and antisense strand, respectively. Line #860 carried a 371 bp excision, verified by Sanger sequencing. **c** PCR of the target region generated a wild-type band for the unedited Sprague-Dawley (SD) sample, two bands for the #860 sample corresponding to the wild-type band and the expected deletion, and no bands for the no-template control (NTC). **d** Western blot of *Disc1* protein from Sprague–Dawley and *Disc1 svΔ2* rat brain samples, performed with DSMS/D22 rabbit anti-DISC1 598-785 antibody. From left to right: Sprague-Dawley female rat, *Disc1 svΔ2* female rat, Sprague-Dawley male rat, and *Disc1 svΔ2* male rat. Western blotting shows absence of an endogenous *Disc1 svΔ2* isoform in male and female animals (red arrow) in *Disc1 svΔ2* male and female samples

The large, N-terminal second coding exon of *Disc1* was selected for targeting via CRISPR/Cas9. Two highly-specific target sequences were utilized to generate a truncated allele that could be followed with simple PCR. Fertilized Sprague-Dawley zygotes were injected with CRISPR/Cas9 reagents, and injected embryos were transferred into pseudopregnant surrogate mothers. Of 23 total F0 animals, 8 founders carried a nonsense, frameshifted and truncated *Disc1* allele as a result of an excision between both target sites. A founder carrying an early translational stop codon at amino acid position 68 was then backcrossed to outbred Sprague-Dawley rats to generate a *Disc1 svΔ2* colony for further characterization (Fig. 1). A Western blot of *Disc1* protein from Sprague-Dawley and *Disc1 svΔ2* rat brain samples was performed with DSMS/D22 rabbit anti-DISC1 598–785 antibody. The DSMS/D22 antibody, recognizes three major isoforms of Disc1 similar to previous published work from the Korth group (one band at ~130 kD and two bands at ~100 kD)^33^. Western blotting further confirmed the truncated allele through the absence of isoform of *Disc1* at 100 kDa (arrow) in both *Disc1 svΔ2* male and female samples.

### *Disc1* svΔ2 harbors broad changes in white matter microstructural integrity

**Fig. 2:**
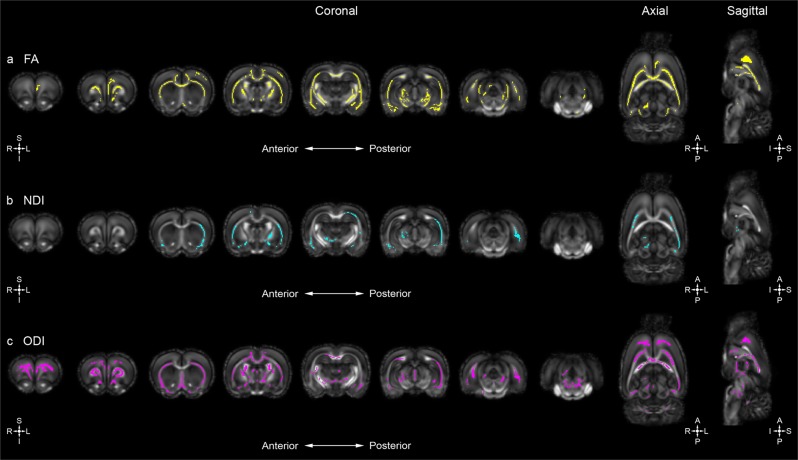
*Disc1 svΔ2* in male rats underlies deficits in white matter microstructural integrity and contributes to global alterations in neurite density and orientation. **a** Whole-brain voxel-wise tract-based spatial reveal significant areas of decreased FA in male *Disc1 svΔ2* rats (*n* = 6) compared to matched controls (*n* = 7) (voxels in yellow). Eight representative coronal sections (left [anterior] to right [posterior]) and single representative axial and sagittal sections reveal significant overlapping regions of decreased FA mainly in the left superior neocortex, external capsule, corpus callosum, internal capsule, and left amygdala. **b** *Disc1 svΔ2* male rats demonstrated significant areas of decreased NDI compared to matched controls (voxels in cyan). Eight representative coronal sections (left [anterior] to right [posterior]) and single representative axial and sagittal sections reveal significant regions of decreased NDI predominantly in the left cerebral hemisphere in the inferior neocortex, external capsule, and right amygdala. **c** *Disc1 svΔ2* male rats demonstrated significant areas of decreased ODI compared to matched controls (voxels in pink). Eight representative coronal sections (left [anterior] to right [posterior]) and single representative axial and sagittal sections reveal significant regions of decreased ODI in the left cerebral hemisphere, and also in a similar spatial distribution in the right hemisphere with decreased ODI spread over a large portion of the superior neocortex, external capsule, and corpus callosum. Some regions of the internal capsule, left amygdala, and left hippocampus also show decreased ODI indices

To explore and characterize the influence of early truncation of the major isoform of *Disc1* on white matter microstructure, ex-vivo whole-brain DTI was performed. Whole-brain voxel-wise tract-based spatial statistics (TBSS) analysis comparing our *Disc1 svΔ2* model to age and sex-matched controls were performed at postnatal day 84 (P84). TBSS identified areas of decreased fractional anisotropy (FA), and increased axial diffusivity (AD), radial diffusivity (RD), and mean diffusivity (MD) (MD = (1/3)(TR); TR = trace diffusivity) following family-wise error correction (*p* *<* .05) in *Disc1 svΔ2* animals across all noted brain regions compared to wild-type controls. Specifically, *Disc1 svΔ2* male rats demonstrate decreased FA mainly in the left superior neocortex, external capsule, corpus callosum, internal capsule, and left amygdala when compared to matched controls (Fig. 2a). The observed FA differences between *Disc1 svΔ2* male rats and controls are accompanied by confluent increased AD, RD, and TR values (Fig. S1). Prior DTI studies describe similar FA decreases in frontal commissural and association fiber tracts in human *DISC1* t(1;11) translocation carriers and in *DISC1* Ser704Cys SNP allele carriers^34,35^. Another study of Ser704Cys *DISC1* allele carriers only observed a convergent trend towards decreased white matter integrity, but did find that fiber tractography-defined anatomical brain networks were significantly less efficient^36^. Additionally of note, prior morphometric neuroimaging studies of human subjects carrying the balanced t(1;11)(q42.1;q14.3) translocation within the *DISC1* gene demonstrated significant reduction of bilateral cortical thickness and reduction of gyrification in prefrontal cortex^37,38^.

**Fig. 3:**
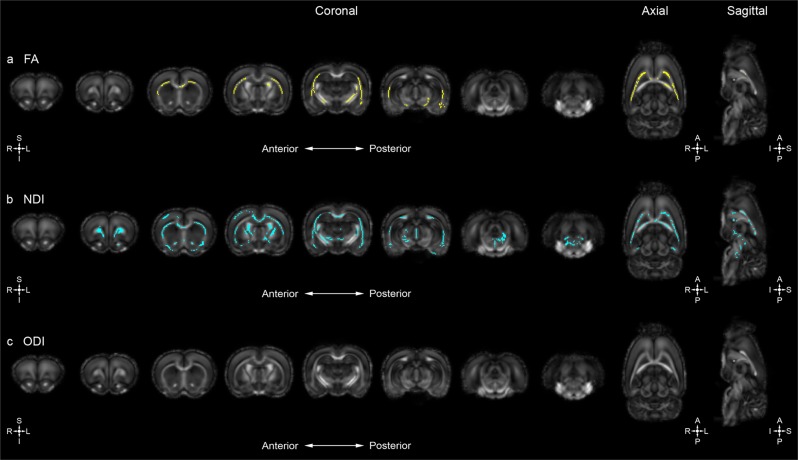
Disc1 svΔ2 in female rats also demonstrates deficits in white matter microstructural integrity and contributes to global alterations inneurite density. **a** Whole-brain voxel-wise tract-based spatial statistics reveal significant areas of decreased FA in female *Disc1 svΔ2* rats (*n* *=* 6) compared to female controls (*n* *=* 6) (voxels in yellow). Eight representative coronal sections (left [anterior] to right [posterior]) and single representative axial and sagittal sections reveal significant regions of decreased FA identified in the left inferior neocortex, external capsule, corpus callosum, and internal capsule. **b** *Disc1 svΔ2* female rats demonstrated significant areas of decreased NDI compared to female controls (voxels in cyan). Eight representative coronal sections (left [anterior] to right [posterior]) and single representative axial and sagittal sections show significant regions of decreased NDI in the right superior neocortex, external capsule, corpus callosum, and right internal capsule. **c** *Disc1 svΔ2* female rats did not demonstrate significant differences in ODI compared to female controls

Sex-specific differences in the distribution of voxel-wise changes in measures of the diffusion tensor are also evident with lower FA values identified in the left inferior neocortex, external capsule, corpus callosum, and internal capsule of female *Disc1 svΔ2* rats when compared to matched controls (Fig. 3a). *Disc1 svΔ2* female rats also show confluent elevated AD, RD, and TR values at the superior neocortex, external capsule, and corpus callosum (Fig. S2). Compared to the *Disc1 svΔ2* males, the extent of the distribution of FA decrease is less pronounced in the areas of overlap including the external capsule, corpus callosum, and internal capsule. It is noteworthy that the aforementioned DTI studies of *Disc1* variants did not include sex as a significant covariate or did not specifically analyze potential sex-specific differences between male and female subjects. Previous analyses from the ENIGMA Schizophrenia DTI Working Group did not observe a significant sex-by-diagnosis interaction for decreases in FA across all major white matter tracts on a region-of-interest (ROI) basis^39^; however, these analyses did not incorporate genetic and environmental factors associated with schizophrenia and thus would not be expected to discover salient sex-specific differences as we find here.

### *Disc1* svΔ2 harbors global alterations in neurite density and orientation

To further explore the role of *Disc1 o*n neural structure and organization, ex-vivo whole-brain neurite orientation, dispersion, and density imaging (NODDI) was also employed. In this first NODDI imaging study applied to a *Disc1 svΔ2* model, voxel-wise TBSS analysis uncovered numerous confluent areas of decreased neurite density index (NDI) and orientation dispersion index (ODI) in the *Disc1 svΔ2* model when compared to age and sex-matched controls (P84). Specifically, *Disc1 svΔ2* males display decreased NDI values, predominantly in the left cerebral hemisphere in the inferior neocortex, external capsule, and right amygdala when compared with matched controls (Fig. 2b). Decreased ODI was seen in a similar pattern in the left cerebral hemisphere, and also in a similar spatial distribution in the right hemisphere with decreased ODI spread over a large portion of the superior neocortex, external capsule, and corpus callosum (Fig. 2c). Some regions of the internal capsule, left amygdala, and left hippocampus also show decreased ODI indices in *Disc1 svΔ2* male rats. As observed in our DTI results, sex-specific differences are also evident in the distribution of voxel-wise change in measures of NDI and ODI. *Disc1 svΔ2* female rats demonstrated decreased NDI in the right superior neocortex, external capsule, corpus callosum, and right internal capsule (Fig. 3b). Conversely, female rats did not show ODI differences when compared with matched controls (Fig. 3c). Previous study of measures of neurite density and orientation dispersion in the context of schizophrenia has been restricted to patients with first episode psychosis. Rae et al. observed significant decreases in NDI across the range of interhemispheric, corticospinal, and association tracts, as well as no significant change in ODI, but the study did not analyze sex differences between male and female TBSS NDI and ODI results^40^. To provide greater context for the literature on sex differences in ODI, past analysis of sex differences in a large-scale UK Biobank study of healthy participants found a global increase in ODI in females compared to males^41^. Other studies^42^, however, have found normal male subjects to harbor a global increase in ODI. Provided these inconsistent findings, a further analysis of sex differences, especially in patients with schizophrenia and other psychiatric illnesses as well as in animal genetic models, will be necessary to characterize patterns, trends, and localization of NDI and ODI differences.

### Early truncation of *Disc1* contributes to significant changes in neural microstructure in salient regions implicated in psychiatric illness

DTI and NODDI analyses sensitively capture microstructural differences in our *Disc1 svΔ2* model across the whole brain when compared to age and sex-matched controls. To further explore the impact of early truncation of *Disc1* in salient regions of the brain implicated in neuropsychiatric illnesses, a ROI analysis was performed. Six regions of interest were *a priori* selected for further analysis: the neocortex, external capsule, corpus callosum, internal capsule, hippocampus, and basal ganglia (including the caudate, putamen, and globus pallidus). Following automated volumetric segmentation of the brain, mean values of both diffusion and neurite indices were computed within each ROI (left and right) for each individual subject for a total of 12 calculated ROIs per subject. *Disc1 svΔ2* male rats had significantly decreased FA values in the right neocortex and in the left basal ganglia ROIs compared to controls, along with significantly increased FA values in the left internal capsule. This finding of increased FA in the left internal capsule is the only instance where, in either TBSS or ROI analysis, *Disc1 svΔ2* animals had higher FA values than wild-type controls. Interestingly, this ROI finding is in contrast to the TBSS results, which indicate a decrease in FA throughout most of the voxels corresponding to the left internal capsule. *Disc1 svΔ2* male rats also demonstrated widespread significant decreases in NDI, with decreases in the bilateral hippocampi, external capsules, basal ganglia, internal capsules, and corpus callosum ROIs. Finally, *Disc1 svΔ2* male rats had significantly reduced ODI in the bilateral basal ganglia and left hippocampus ROIs (Table 1). Stricter analyses controlling for multiple comparisons (false discovery rate with the Benjamini–Hochberg procedure with a FDR set to .05) sustained the significant difference findings for FA in the right neocortex, NDI in the right basal ganglia, bilateral hippocampus, external capsule, internal capsule, and corpus callosum, and ODI in the right basal ganglia and left hippocampus. *Disc1 svΔ2* female rats demonstrated significantly decreased FA in the right corpus callosum and left internal capsule and neocortex ROIs. Significant reductions in NDI were observed in bilateral hippocampus and corpus callosum, right internal capsule, and left basal ganglia and neocortex. The only *Disc1 svΔ2* female rat ROI with significantly lower ODI was right neocortex (Table 1). Of these significant results, the right corpus callosum decrease in NDI was significant after applying the FDR procedure.

**Table 1:** *Disc1 svΔ2* contributes to significant changes in neural microstructure in salient regions implicated in psychiatric illness

DTI measure	Hemi.	ROI	Mean (±SEM)		*p*-value	Mean (±SEM)		*p*-value
			Control male	Disc1 ΔN68/ΔN68 male	Male	Control female	Disc1 ΔN68/ΔN68 female	Female
FA	Right	HC	.31328 (±.00027)	.31374 (±.00055)	.772	.31154 (±.00040)	.31217 (±.00038)	.652
		EC	.45444 (±.00032)	.45761 (±.00068)	.131	.45583 (±.00101)	.45581 (±.00077)	.993
		BG	.29129 (±.00036)	.29081 (±.00037)	.716	.29213 (±.00045)	.29202 (±.00040)	.945
		IC	.44397 (±.00044)	.44442 (±.00150)	.912	.44048 (±.00241)	.44777 (±.00104)	.296
		NC	.28768 (±.00010)	.28563 (±.00015)	.001*	.28781 (±.00029)	.28816 (±.00019)	.696
		CC	.52396 (±.00024)	.52346 (±.00056)	.749	.51810 (±.00059)	.52261 (±.00028)	***.024***
	Left	HC	.32838 (±.00024)	.32878 (±.00048)	.772	.33096 (±.00085)	.32766 (±.00025)	.178
		EC	.42046 (±.00048)	.41847 (±.00112)	.532	.41288 (±.00164)	.41917 (±.00093)	.209
		BG	.30576 (±.00031)	.30126 (±.00049)	***.013***	.30399 (±.00042)	.30368 (±.00038)	.831
		IC	.55447 (±.00059)	.56802 (±.00182)	*.* ***027***	.57168 (±.00118)	.56001 (±.00164)	***.043***
		NC	.28813 (±.00034)	.28689 (±.00037)	.076	.28946 (±.00013)	.28743 (±.00028)	**.033**
		CC	.57720 (±.00060)	.57595 (±.00051)	.346	.57198 (±.00072)	.57341 (±.00105)	.658
NDI	Right	HC	.28941 (±.00340)	.23560 (±.00206)	**<** .001*	.23103 (±.00517)	.30632 (±.00796)	***.011***
		EC	.30236 (±.00541)	.23182 (±.00201)	.001*	.27036 (±.01174)	.29071 (±.00652)	.554
		BG	.28386 (±.00481)	.22498 (±.00248)	.003*	.22495 (±.00523)	.29500 (±.01196)	.065
		IC	.38430 (±.00245)	.30675 (±.00613)	.002*	.29660 (±.00560)	.35418 (±.00714)	***.028***
		NC	.33364 (±.00438)	.30249 (±.00395)	.052	.31497 (±.00439)	.34905 (±.00744)	.145
		CC	.41147 (±.00386)	.31803 (±.00591)	**<** .001*	.27198 (±.00352)	.38752 (±.00487)	**<** .001*
	Left	HC	.28968 (±.00207)	.23452 (±.00275)	**<** .001*	.24458 (±.00589)	.29333 (±.00656)	***.048***
		EC	.29344 (±.00187)	.24496 (±.00186)	**<** **.** 001*	.24774 (±.00552)	.29113 (±.00720)	.082
		BG	.28773 (±.00294)	.23324 (±.00257)	**<** ***.001***	.23410 (±.00451)	.30759 (±.01068)	***.037***
		IC	.44053 (±.00370)	.33749 (±.00524)	**<** .001*	.33155 (±.00351)	.36690 (±.01050)	.239
		NC	.35425 (±.00287)	.32002 (±.00379)	***.015***	.31738 (±.00462)	.35627 (±.00471)	***.037***
		CC	.43527 (±.00341)	.34018 (±.00519)	**<** .001*	.30457 (±.00596)	.38664 (±.00506)	***.002***
ODI	Right	HC	.20010 (±.00465)	.17447 (±.00389)	.128	.18642 (±.00829)	.24960 (±.01015)	.079
		EC	.18074 (±.00363)	.15364 (±.00418)	.080	.19363 (±.01058)	.19580 (±.00838)	.949
		BG	.25877 (±.00590)	.19671 (±.00366)	.007*	.20442 (±.00696)	.27633 (±.01724)	.161
		IC	.17413 (±.00344)	.15744 (±.00956)	.530	.19334 (±.01802)	.21625 (±.01752)	.718
		NC	.23216 (±.00488)	.20261 (±.00412)	.100	.20200 (±.00440)	.27158 (±.00677)	***.007***
		CC	.12962 (±.00245)	.14678 (±.01170)	.582	.19095 (±.01923)	.13683 (±.00527)	.312
	Left	HC	.19648 (±.00173)	.16911 (±.00252)	.005*	.20201 (±.00865)	.22323 (±.00762)	.470
		EC	.18265 (±.00260)	.16419 (±.00563)	.268	.18702 (±.01109)	.19980 (±.01016)	.736
		BG	.24587 (±.00485)	.20683 (±.00245)	***.024***	.22083 (±.00696)	.28836 (±.01386)	.117
		IC	.13344 (±.00378)	.15946 (±.01595)	.544	.24072 (±.02132)	.27034 (±.01301)	.641
		NC	.24230 (±.00207)	.22947 (±.00500)	.371	.25140 (±.00408)	.25701 (±.00814)	.809
		CC	.12929 (±.00209)	.14124 (±.01569)	.770	.20886 (±.02381)	.13412 (±.00450)	.260

All values are mean ± s.e.m. Units of measure for FA, NDI, and ODI are [10^−3^ mm^2^/s]. Bolded and italicized *p*-values are statistically significant. Starred *p*-values are statistically significant after controlling the false discovery rate with the Benjamini-Hochberg procedure (false discovery rate = .05). Regions of interest (ROIs) correspond to ROIs derived from the P72 UNC Atlas

For control male samples, *n*  = 7. For all other sample groups, *n* = 6

*Hemi*. hemisphere, *FA* fractional anisotropy, *NDI* neurite density index, *ODI* orientation dispersion index, *HC* hippocampus, *EC* external capsule, *BG* basal ganglia, *IC* internal capsule, *NC* neocortex, *CC* corpus callosum

For *Disc1 svΔ2* male rats, a decrease in NDI was observed in the bilateral hippocampi, internal capsules, corpus callosum, and basal ganglia ROIs, all of which did not display significant differences on a voxel-wise basis in our TBSS analysis. Conversely, TBSS analysis uncovered a global significant decrease in ODI that is not reflected in the ROI analysis outside of the hippocampus and basal ganglia. These findings, while at first contradictory, reinforce the complementary information that is found on both a voxel-wise level as well as a more biologically-relevant segmentation of the neuroimaging data. An exemplar of this can be seen in the NODDI analysis of the internal capsule. On a voxel-wise basis, we are able to appreciate a significant decrease in ODI in *Disc1 svΔ2* male rats in the internal capsule with no voxel-wise changes in NDI. Subsequent ROI analysis reveals the converse—we find significantly decreased NDI in the internal capsule with no appreciable change in ODI. These seemingly divergent results rather reflect the level of results that interest us as voxel-level analyses detail local microstructural changes in contrast to ROI analyses that reveal important neuroanatomical alterations.

### Measures of prepulse inhibition are not significantly impacted in our *Disc1 svΔ2* model

**Fig. 4:**
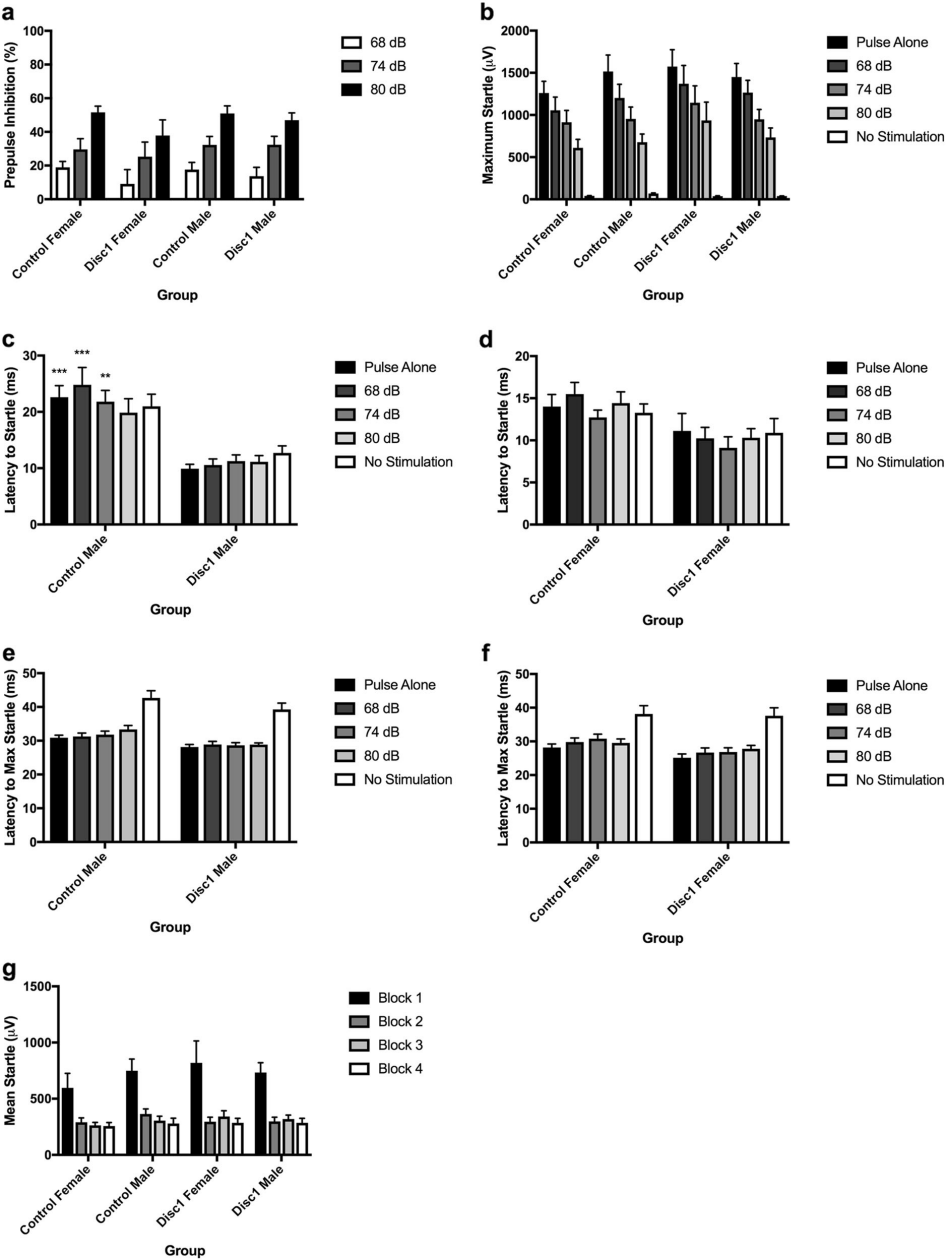
Measures of prepulse inhibition are not significantly impacted by early truncation of *Disc1*. **a** Quantification of prepulse inhibition in P84 *Disc1 svΔ2* rats and wild-type control rats. **b** Quantification of startle response magnitude in P84 *Disc1 svΔ2* rats and wild-type control rats. **c** Quantification of latency from trial onset to startle response for male wild-type and male *Disc1 svΔ2* rats. **p-value < .01; ***p-value < .001, by three-way ANOVA with Tukey’s post hoc tests. **d** Quantification of latency from trial onset to startle response for female wild-type and female *Disc1 svΔ2* rats. **e** Quantification of latency from trial onset to maximum startle response for male wild-type and male *Disc1 svΔ2* rats. **f** Quantification of latency from trial onset to maximum startle response for female wild-type and female *Disc1 svΔ2* rats. **g** Quantification of habituation to startle-alone trials. Error bars denote mean ± s.e.m. For control male group, *n* *=* 24. For control female group, *n* = 18. For *Disc1 svΔ2* male and female groups, *n* = 15

Early truncation of *Disc1* imparts significant changes in white matter structural integrity and neurite density and orientation globally throughout the brain and in salient neuroanatomical regions implicated across a broad spectrum of psychiatric illness. We next asked if these striking microstructural changes would manifest at the systems-level with changes in animal behavior. Overall, *Disc1 svΔ2* animals did not demonstrate significantly differences in prepulse inhibition (PPI) when compared to control animals (Fig. 4a). As expected with PPI, ANOVA revealed a significant main effect of prepulse intensity (*F*(2, 134) = 105.70, *p* *<* .0001) a standard parametric feature of PPI where increasing prepulse intensities elicit higher levels of PPI^43^. There were not, however, other significant main or interaction effects between prepulse intensity, sex or genotype. Latency data was analyzed next. ANOVA of latency to startle revealed a significant effect of genotype (Control > *Disc1 svΔ2*; *F*(1, 67) = 22.018, *p* < .0001) and sex (Male > Female; (*F*(1, 67) = 12.158, *p* < .001), and a significant interaction of gender × genotype (*F*(1, 67) = 5.131, *p* < .05). There were no other significant main effects or interaction effects. Post-hoc analyses found that for the control male to *Disc1 svΔ2* male comparison, control males had significantly longer latencies to startle onset on the pulse-alone, 68 dB, and 74 dB trial types (*p* *<* .001, *p* *<* .0001, *p* *<* .05, respectively) (Fig. 4c). We next turned our attention to analyses of maximum startle and latency to maximum startle. In the analysis of maximum startle, ANOVA revealed a significant main effect of trial type on maximum startle (F(4,272) = 141.539, *p* *<* .0001) with no other significant main effects or interaction effects, indicating that the maximum startle response does not differ for *Disc1 svΔ2* animals or controls across the entire experiment (Fig. 4b). ANOVA of latency to maximum startle revealed a significant effect of genotype (Control > *Disc1 svΔ2*; *F*(1, 93) = 9.367, *p* *<* .01), sex (Male > Female; *F*(1, 93) = 9.367, *p* *<* .01), and trial type (*F*(4, 272) = 53.756, *p* *<* .0001) with no significant interaction effects. The latency-to-startle onset and latency-to-startle peak are related measures of the rapidity for which a startling stimulus elicits the startling response. In schizophrenia patients, latency-to-startle is significantly increased. In our review of literature involving behavioral measures in murine *Disc1* mutations, we have found a wide range of results regarding PPI deficits and have not encountered any reports on latency to startle. Our finding of significantly reduced latency to startle in the *Disc1 svΔ2* rats compared to controls contradicts findings in humans with schizophrenia, but does not have a point of comparison from other murine models. Finally, habituation data was analyzed, where there were no significant main effects or interaction effects, indicating that habituation to the repeated startle stimulus across the session did not differ between *Disc1 svΔ2* rats and controls (Fig. 4g).

## Discussion

In this study, we present a novel *Disc1 svΔ2* rat model of psychiatric illness and demonstrate the significant impact early truncation of *Disc1* imparts along multiple quantitative neuroimaging measures of neural structure. Our neuroimaging studies reveal and clarify both the axonal contributions (DTI) and synaptic contributions (NODDI) to the psychiatric disease state and quantitatively capture the microstructural changes that accompany an important gene variant implicated in several psychiatric illnesses. The generation of a *Disc1 svΔ2* rat model was chiefly motivated by our understanding of DISC1 as a major molecular scaffold protein interacting with GSK3*β*, NDEL1, LIS1, PDE4, KAL7, TNIK, and others in multiple neuronal processes, thus placing it at the molecular intersection of schizophrenia and numerous other major psychiatric illnesses^44,45^. With DISC1 widely expressed throughout the brain, particularly in the cortex, hippocampus, hypothalamus, cerebellum, and brain stem, the *Disc1* genetic model emerges as a natural and convenient entry point to explore the neurostructural features and consequences of the presence of short variants of *Disc1* across several psychiatric illnesses and concomitantly, with the generation of a short variant genetic *Disc1 svΔ2* animal model, provide a new platform to explore the biological role of *Disc1* in the neuropathogenesis of psychiatric illness. As discussed in Tomoda et al., a *Disc1* animal model enables the evaluation of circuit-level disturbances, comparison to functional and structural imaging data from patients with mental disorders, and potential validation of biomarkers useful for diagnosis and targets for therapeutic intervention^16,17^.

Our *Disc1 svΔ2* model recapitulates many of the major diffusion tensor neuroimaging findings seen in clinical populations of schizophrenia including decreased FA in the corpus callosum, left frontal neocortex, and in the deep gray nuclei. As DISC1 interacts with a class of proteins (e.g., MAP1A, MIPT3, ATF4/5, and NUDEL) that associate with microtubules and their associated complexes during key developmental time points in neuronal migration and patterning^46,47^ and as DISC1 microtubule-associated processes are also involved in corticogenesis in vivo during the radial migration of neurons during cortical development^48^, we would speculate that perturbations to these tightly orchestrated processes would produce the deficits in white matter structural integrity observed in our diffusion tensor study. Our diffusion tensor results also highlight the sex-specific effect of early truncation *Disc1*; whereas both sexes demonstrate significant changes in FA in the salient aforementioned regions, male animals demonstrate a greater quantity of change that is more diffusely distributed throughout the brain. While qualitative, nonetheless, these findings of greater and more diffuse decreased white matter microstructural integrity in male animals intriguingly suggests a structural predisposition to the psychiatric disease state and dovetails with the clinically observed increased prevalence of male psychopathy, particularly in schizophrenia.

Expanding on the work of DTI and other single-compartment diffusion weighted imaging techniques, multi-compartment diffusion weighted imaging techniques such as NODDI allow us to interrogate tissue-specific microstructural features that are at once quantitative and, importantly, clinically feasible^49^. To generate greater tissue specificity than standard DWI techniques such as DTI, NODDI employs a model-based strategy designed to measure water diffusion arising from distinct tissue compartments^49,50^. These compartments include the intra-neurite compartment (axons and dendrites), the extra-neurite compartment (glial cells), and cerebrospinal fluid (CSF). An advantage of multi-compartmental models such as NODDI over DTI is the ability of NODDI to sensitively capture changes in neurite density (neurite density index, NDI) and orientation (orientation dispersion index, ODI), two microstructural features that are occult on standard diffusion tensor imaging and important microstructural features that are representative of underlying synaptic density and organization. A salient example of the added utility of using NODDI is demonstrated in Colgan et al., where a tau pathology murine model demonstrated significantly higher neurite density in neocortex than in wild-type controls, correlating with the degree of tau burden, in the absence of significant FA and MD findings^51^. As DISC1 serves to regulate the development of synaptic growth and the organization of trans-synaptic structures and functions, it would thus be predicted to impact neuroimaging measures of neurite density and orientation^44,45,52–54^. As anticipated, our *Disc1 svΔ2* model harbors decreased neurite density in numerous salient regions of the brain including the hippocampal formation, the basal ganglia, and the neocortex, consistent with previous studies of both dysmorphic and decreased dendritic density and arborization as seen in models of both *Disc1* under- and overexpression^55,56^. Just as with our DTI results, our NODDI findings in our *Disc1 svΔ2* model also reflect sex-specific differences in neural microstructure. Male animals display more global changes in NDI when compared to females as evidenced on both a voxel-wise and ROI basis. Interestingly, females harboring truncated *Disc1* demonstrate little to no change in measures of ODI, whereas male animals demonstrate striking changes in ODI in both TBSS and ROI analyses. As noted in our DTI analysis, these findings of greater and more diffuse decreased NDI and ODI matter microstructural integrity in male animals again suggests a structural predisposition to the psychiatric disease state. In particular, that females demonstrate a similar extent and distribution of decreased NDI but little to no change in ODI suggests that like their male counterparts, females harbor decreased neurite density but are able to maintain the overall orientation of their remaining neurites as opposed to males who display decreased synaptic density concomitantly with decreased (less complex) neurite orientation.

A major difficulty and limitation in the development of animal models towards understanding mental illness has been the ability to recapitulate the full range of phenotypes that make up even a single disorder, let alone a group of disorders. In the absence of these more-complete models, the use of models for which some features do accurately recreate important aspects of the biology of a group of disorders will continue to hold importance. PPI deficits, especially in a *Disc1* model such as ours, would help to provide content validity as one of the quantitative endophenotypes associated with schizophrenia^57^. At first glance, our findings do not agree with previous studies showing PPI deficits in rodent *Disc1* genetic models^18,19^. Nevertheless, the degree of genetic mutation as well as the behavioral testing parameters used in our study versus these previous reports vary significantly. Moreover, there are recent reports with *Disc1* point mutation models indicating that this manipulation does not disrupt PPI^58,59^. Importantly, our study for the first time indicates that other startle plasticity parameters besides PPI may be of relevance in the study of the behavioral phenotypes associated with DISC1, as we found significant alterations in latency to startle. No other *Disc1* animal models have considered these additional features of PPI, and their clinical significance remains to be further established. The finding that global PPI deficits were not observed, but these subtler alterations in other startle plasticity measures were seen, may indicate that our *Disc1* model acts to make subjects more ‘vulnerable’ to other factors that could then lead to a more pronounced PPI effect, perhaps such as low-dose psychotomimetic drugs or stress exposure. As noted earlier, the broad range of genetic manipulations underlying previously reported *Disc1* models, variation in behavioral phenotypic expression is thus expected. Another hypothesis regarding our PPI findings is that our *Disc1 svΔ2* model in rats may represent a PPI phenotype comparable to the wild-type and that subsequent environment challenges such as early-life stress would uncover and unmask the PPI deficit, consistent with numerous gene-environment models of neuropsychiatric illness^60–64^.

We have demonstrated the generation of the first biallelic *Disc1 svΔ2* rat model, which recapitulates major DTI measures of decreased white matter structural integrity observed in human individuals and for the first time, demonstrates the contribution of *Disc1* to microstructural features of neurite density and orientation. Sex-specific differences in neural structure—on both DTI and NODDI—clearly illustrate the greater extent of microstructural change present in male animals mirroring the greater male sex-specific predilection towards the development of psychiatric illness that is observed clinically. Further investigation of the combinatorial effects of our *Disc1 svΔ2* genetic model and environmental challenge on measures of neural microstructure and animal behavior including PPI and other phenotypic measures of anxiety, sociability, and working memory represent an important next step. These efforts will contribute to our understanding of the degree of genetic susceptibility imparted by *Disc1* and the degree to which the environmental milieu can exacerbate or ameliorate the psychiatric disease state.

## Supplementary information

Supplementary Material.

## Data Availability

All data that support the findings of this study are available from the corresponding author upon request.
