# Supplementary material for: Sex-specific deficits in neurite density and white matter integrity are associated with targeted disruption of exon 2 of the Disc1 gene in the rat
Source: Transl Psychiatry. 2019 Feb 11;9:82. doi: 10.1038/s41398-019-0429-2 (PMC6370885; doi:10.1038/s41398-019-0429-2)
Supplement: Supplementary file 1 — Supplementary Material. [file 41398_2019_429_MOESM1_ESM.docx]

**Sex-specific deficits in neurite density and white matter integrity are associated with targeted disruption of exon 2 of the *Disc1* gene in the rat**

**Supplementary Material**

**Figure S1**

***Disc1 svΔ2* in male rats underlies deficits in white matter microstructural integrity across DTI indices and contributes to global alterations in neurite density and orientation**


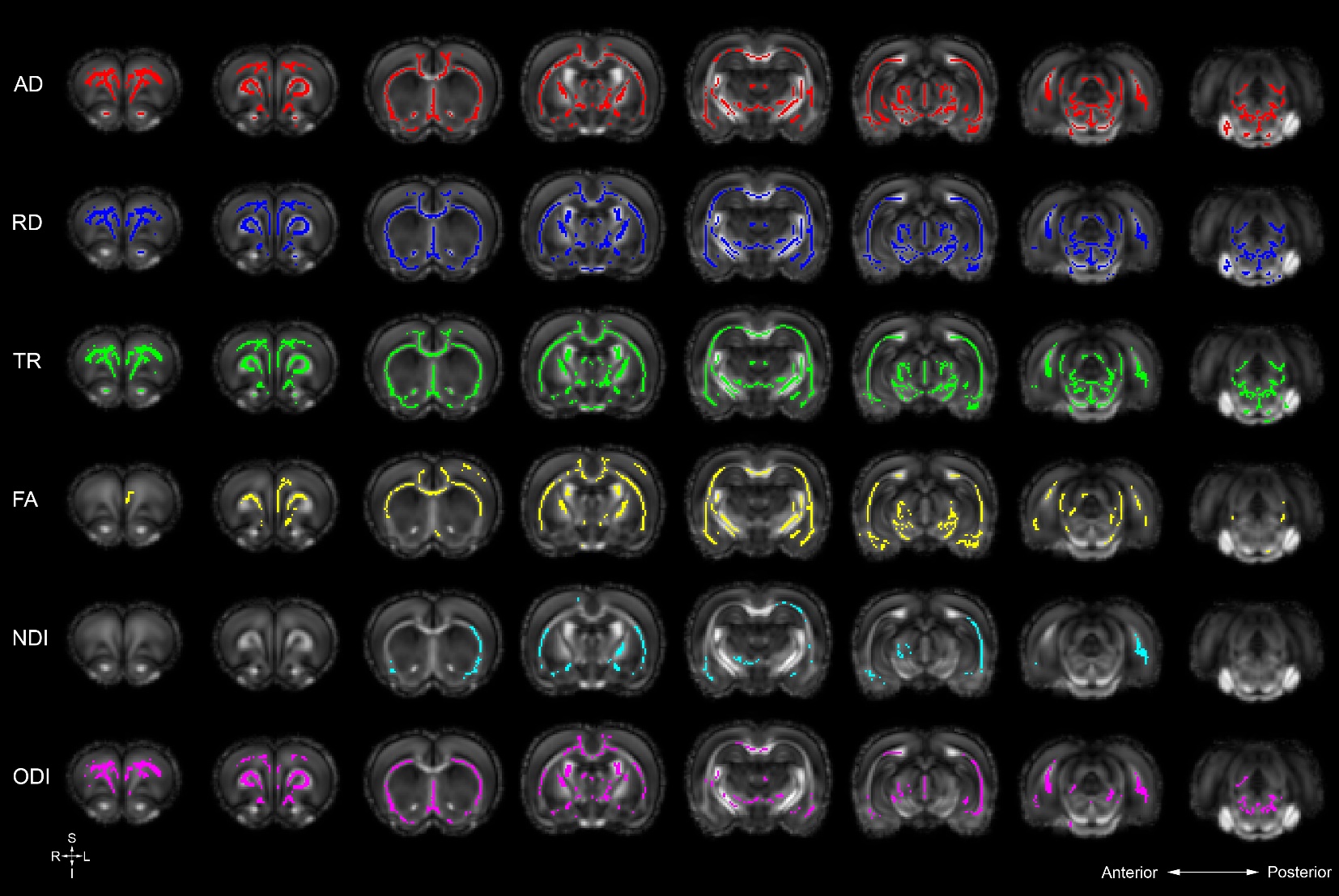


Whole-brain voxel-wise tract-based spatial statistics (TBSS) uncovered statistically significant areas of difference in male *Disc1 svΔ2* rats (*n* = 6) compared to male controls (*n* = 7) in axial diffusivity (AD), radial diffusivity (RD), trace (TR; trace: mean diffusivity [MD] x3), fractional anisotropy (FA), neurite density index (NDI), and orientation dispersion index (ODI) at postnatal day 84 [P84]. Eight representative coronal sections (left [anterior] to right [posterior]) are presented for each measure. Increased AD, RD, and TR values are observed in *Disc1* male rats when compared to matched controls; along with decreased FA, NDI, and ODI indices. AD = red; RD = blue; TR = green; FA = yellow; NDI = light-blue; ODI = magenta. Family-wise error corrected, *p* < .05.

**Figure S2**

***Disc1 svΔ2* in female rats underlies deficits in white matter microstructural integrity across DTI indices and contributes to global alterations in neurite density and orientation**


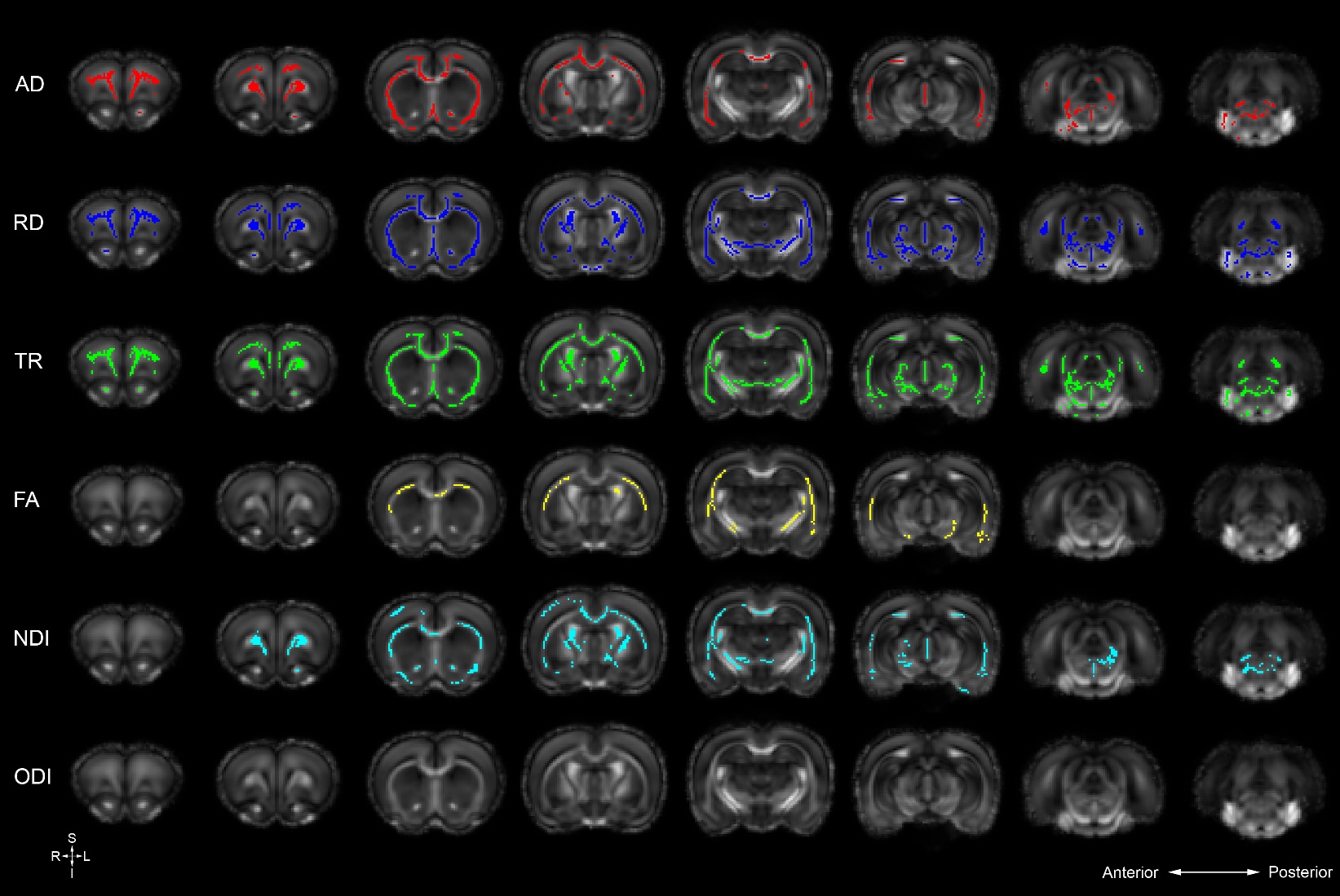


Whole-brain voxel-wise tract-based spatial statistics (TBSS) uncovered statistically significant areas of difference in female *Disc1 svΔ2* rats (*n* = 6) compared to female controls (*n* = 6) in axial diffusivity (AD), radial diffusivity (RD), trace (TR; trace: mean diffusivity [MD] x3), fractional anisotropy (FA), and neurite density index (NDI) at postnatal day 84 [P84]. Eight representative coronal sections (left [anterior] to right [posterior]) are presented for each measure. Increased AD, RD, and TR values are observed in *Disc1* female rats when compared to matched controls; along with decreased FA and NDI indices. AD = red; RD = blue; TR = green; FA = yellow; NDI = light-blue; ODI = magenta. Family-wise error corrected, *p* < .05.
